# Extending the Shelf Life of Raw Milk and Pasteurized Milk with Plantaricin FB-2

**DOI:** 10.3390/foods12030608

**Published:** 2023-02-01

**Authors:** Yajuan Li, Peifang Weng, Zufang Wu, Yanan Liu

**Affiliations:** Department of Food Science and Engineering, Ningbo University, Ningbo 315211, China

**Keywords:** raw milk, pasteurized milk, plantaricin FB-2, shelf life

## Abstract

Raw milk and pasteurized milk are characterized by a short shelf life, and drinking expired raw milk and pasteurized milk causes illness. In the study, Plantaricin FB-2 (extracted from *Lactiplantibacillus plantarum* FB-2) was added to liquid milk. By evaluating the microbial growth, acidity changes, protein content, and sensory changes in raw milk and pasteurized milk during storage, it was found that when Plantaricin FB-2 was added at 0.4 g/kg, the shelf life of raw milk was extended by 3 days (7 days if not added). The shelf life of pasteurized milk with Plantaricin FB-2 was extended to 31 days (25 days in the control group), and the optimal amount was 0.3 g/kg. This confirmed that Plantaricin FB-2 can effectively prolong the shelf life of raw and pasteurized milk. This study provides valuable information for the application of bacteriocins produced by *Lactiplantibacillus plantarum* in raw milk and pasteurized milk to improve their shelf life.

## 1. Introduction

Food safety is a challenging and persistent problem worldwide, which has attracted great social attention due to multiple outbreaks of foodborne diseases. Foodborne diseases are mainly caused by the presence of pathogenic microorganisms in food, which can pose a threat to human health. Milk has high nutrient content and can easily provide a favorable environment for the growth of spoilage and pathogenic microorganisms [1]. Consumption of improperly preserved or expired raw and pasteurized milk may pose health risks due to contamination by various pathogenic microorganisms, which can lead to a series of symptoms such as abdominal pain and diarrhea, and even death in severe cases [2].

Raw milk has a complex microbiota, including bacteria and fungi. Among them, the bacteria mainly include, *Pseudomonas*, *Acinetobacter*, *Aeromonas* spp. [3], *Escherichia* [4], *Lactococcus*, *Lactobacillus*, *Leuconostoc* [5], *Streptococcus, Staphylococcus* [6], and so on. Fungi include molds and yeasts. The molds mainly include *Acremonium* and *Mucor* [7]. Pasteurized milk is raw milk that has been pasteurized. During storage, it is easily polluted by *Pseudomonas* [8], *Escherichia* [9], *Staphylococcus* [10], and molds and yeasts. The molds mainly include *Aspergillus* [11].

At present, bacteriocins are used as new bioderived bacteriostatic agents. It prevents food from spoiling by inhibiting the growth of foodborne pathogens, thus extending shelf life with minimal impact on food flavor. Plantaricin UG1 applied to pasteurized milk can effectively inhibit the growth and reproduction of *Bacillus cereus* [12]. Plantaricin BM-1 can inhibit the growth and reproduction of *Listeria monocytogenes* in cooked ham [13]. Plantaricin GZ1-27 combined with chitosan had a biological preservative effect on chilled pork [14]. Plantaricin EmF combined with chitosan effectively inhibited *Listeria monocytogenes* and extended the shelf life of beef [15]. These applications have shown that plantaricin has a good inhibitory effect on common foodborne pathogens and has great potential in food preservation.

In this study, Plantaricin FB-2 extracted from *Lactiplantibacillus plantarum* FB-2 was added to raw milk and pasteurized milk at different concentrations. The microorganism, methylene blue reduction reaction, acidity, and protein content of the samples during storage were analyzed, combined with sensory evaluation, to determine the effect of Plantaricin FB-2 on the shelf life. In particular, in the microbial analysis, the mixed effects model was used for microbial prediction. This study confirmed that Plantaricin FB-2 is an effective preservative, which can provide a reference for the prediction of the microbial growth in raw and pasteurized milk in the future, and expand the application of plantaricin.

## 2. Materials and Methods

### 2.1. Raw Milk and Pasteurized Milk Sample

In this work, two kinds of fluid milk were analyzed: raw milk (Ningbo Milk Group Co., Ltd., Ningbo, China) and pasteurized milk (Bright Dairy and food Co., Ltd., Shanghai, China). The samples were newly collected or purchased on the day of the experiment started.

### 2.2. Preparation of Plantaricin FB-2

Plantaricin FB-2 was extracted from *Lactiplantibacillus plantarum* FB-2 (preserved at food biotechnology laboratory of Ningbo University, Ningbo, China). It has been proved to have antimicrobial activity. The activated *Lactiplantibacillus plantarum* FB-2 was fermented for 20 h. The supernatant was extracted by centrifugation (4 °C, 6000 rpm, 10 min). The supernatant was precipitated with ammonium sulfate (90% saturation) at 4 °C. The crude extract was taken out from the dialysis bag and freeze-dried for later use.

### 2.3. Experimental Design

Under sterile conditions, Plantaricin FB-2 was dissolved in sterile saline and sterilized by filtration with a 0.22 μm microporous membrane. The milk sample was added at different concentrations, i.e., C_1_ = 0.1 g/Kg, C_2_ = 0.2 g/Kg, C_3_ = 0.3 g/Kg, C_4_ = 0.4 g/Kg, and C_5_ = 0.5 g/Kg. In the control group, normal saline was added to the sample. The raw milk was analyzed immediately after unpacking for each day during storage at 4 °C, while the pasteurized milk was analyzed immediately after unpacking every 3 days during storage at 4 °C.

### 2.4. Bacterial Counts

Microbiological enumerations were performed on raw and pasteurized milk samples. A 25 g sample was diluted with 225 mL of peptone salt solution consisting of 8.5 g/L NaCl, and then spread by the 10-fold dilution method. The cultivation methods of different kinds of microorganisms were shown in Table 1 [16,17,18].

### 2.5. Microbial Growth Model Prediction

The Q test was performed on the measured values of colony, and the suspicious value was eliminated. The confidence level was 90%. Repeated measurements from 3 samples were used for the number of colonies for each microorganism. The dependent variable was the number of colonies per microorganism, adjusting for a mixed linear model that included the fixed effects for group ((GRP) C_0_, C_1_, C_2_, C_3_, C_4_ and C_5_), time ((TIME) day 0 to 12 days in raw milk and day 1 to 37 days in pasteurized milk as replicates measurement), and the random effects ((L) the variation in sample individuals over time). Among them, the categorical variables were groups and time, the continuous variable was the number of colonies per microorganism, the measurement range was 0–10^15^ CFU/mL, and the probability distribution followed a logarithmic normal distribution. The data analysis was performed using PROC MIXED of SAS package, version 9.4, fitting a covariance structure of compound symmetry (CS). The linear expression of the model was:

(1) $$y_{ij}=\mu +GRP_{j}+TIME_{ij}+GRP_{j}\times TIME_{ij}+L_{ij}+\varepsilon_{ij}$$

in which the abbreviations indicated $y_{ij}$ = the logarithmic values of the number of colonies per microorganism, μ = overall mean, $GRP_{j}$ = groups fixed effects, $TIME_{ij}$ = fixed effect of sampling days (repeated measures), $GRP_{j}\times TIME_{ij}$ = interactive effects of groups and time, $L_{ij}$ = random effects between samples, and $\varepsilon_{ij}$ = residual random effects. A comparison of least square means between treatments were performed using all pairwise differences.

### 2.6. Methylene Blue Reduction Reaction

Methylene blue aqueous solution with a concentration of 0.01 g/300 mL was prepared 10 mL milk was absorbed into a sterilized test tube, heated to 38 °C in the water bath, added 1 mL of methylene blue solution and mixed well. The sample was placed in a water bath (maintained at 38 °C), the fading was observed every 30 min, and the fading time of each sample was recorded.

### 2.7. Changes in Acidity

The acidity (°T) of milk was titrated by sodium hydroxide solution, C_(NaOH)_ = 0.1 mol/L, and phenolphthalein was used as indicator. The acidity of a sample can be determined by titrating the amount of NaOH (mL) consumed by 100 mL of milk.

### 2.8. Changes in Protein Content

A BCA kit (TransGen Biotech, Beijing, China) was used for the determination of protein content.

### 2.9. Sensory Evaluation

In this study, five professionals were selected to evaluate raw and pasteurized milk on five indicators, including odor, color and luster, texture, boiling state, and mouthfeel. The specific evaluation criteria were shown in Table 2. ANOVA was applied to the descriptive analysis. Significant difference among univariate mean values were determined for each data set using Fisher’s Least Significant Difference (LSD). The significant descriptive analysis attributes were analyzed by applying PCA to the evaluation index. The data analyses and PCA were performed using python 3.8. The LSD test was performed using R 2022.07.1.

### 2.10. Data Analysis

The experiment was repeated three times. The ANOVA at *p* ≤ 0.05 level and least significant difference (LSD) tests were used to statistically analyze the means of triplicate data using SPSS (V.26).

## 3. Results and Discussion

### 3.1. Microbiological Analyses

There is a complex microbiome in milk, which has an important influence on the shelf life of milk. The most important of these are psychrotrophic bacteria which are the dominant spoilage bacteria of milk. Psychrotrophic bacteria can proliferate in large numbers during refrigeration and cause milk spoilage by producing extracellular lipases and proteases [19]. In addition, studies have shown that during storage of dairy products, *Staphylococcus aureus* and *Enterobacteriaceae* can increase [20,21,22], and the reproduction of mold and yeast can also shorten the shelf life of dairy products [7].

**Figure 1:**
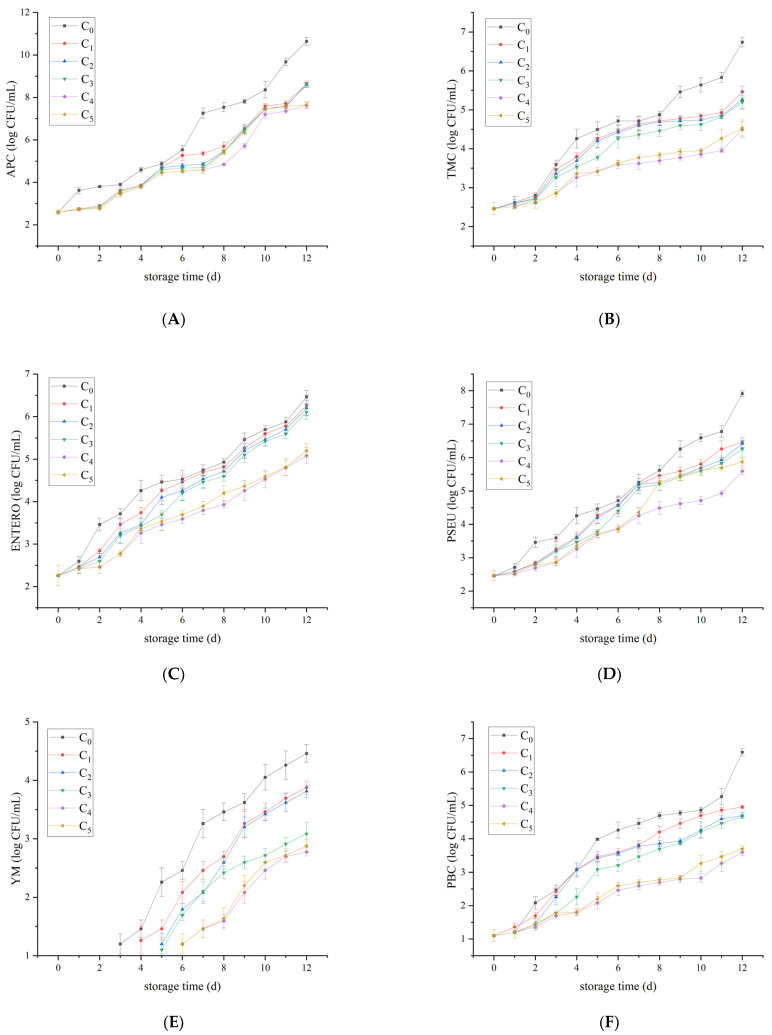

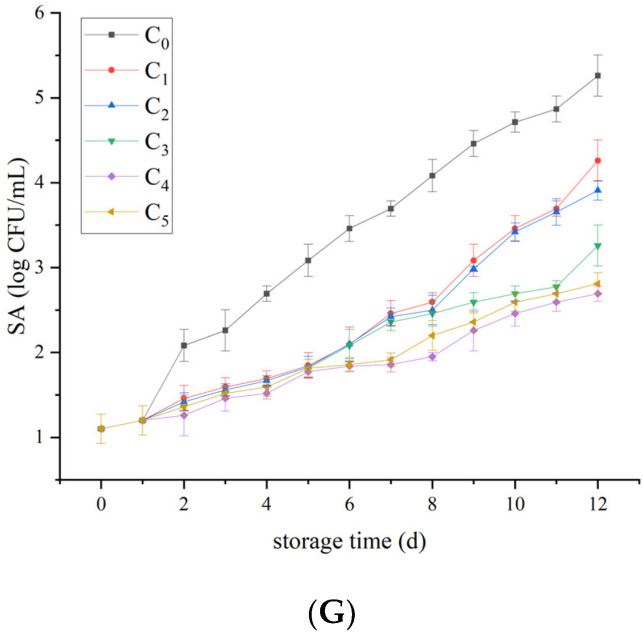
Microbiological counts of the raw milk. (**A**) APC, (**B**) TMC, (**C**) ENTERO, (**D**) PSEU, (**E**) YM, (**F**) PBC, and (**G**) SA. Plantaricin FB-2 concentration was C_1_ = 0.1 g/kg, C_2_ = 0.2 g/kg, C_3_ = 0.3 g/kg, C_4_ = 0.4 g/kg, and C_5_ = 0.5 g/kg, and C_0_ was the control group, and the same amount of normal saline was added.

In China, the national food safety standard stipulates that APC in raw milk should be ≤2 × 10^6^ CFU/mL. It can be seen from Figure 1 that on the 7th day, the APC of the control group was 1.82 × 10^7^ CFU/mL and began to deteriorate. On the 10th day, the C_4_ group was 1.58 × 10^7^ CFU/mL and began to deteriorate. On day 12, there was no significant difference between the C_4_ and the C_5_ groups, but it was significantly different from other groups (*p* < 0.05). On day 12, PSEU reached 8.13 × 10^7^ CFU/mL in the control group and 3.89 × 10^5^ CFU/mL in the C_4_ group. Psychrotrophic bacteria, *Enterobacteriaceae*, *Pseudomonas,* and *Staphylococcus aureus* had similar growth and reproduction rules. It is worth noting that in the beginning, raw milk did not contain YM. The control group began to appear YM on the 3rd day, while the C_4_ and C_5_ groups began to appear on the 6th day. On day 12, YM in the control group reached 2.8 × 10^4^ CFU/mL, and the C_4_ group was only 5.89 × 10^2^ CFU/mL. There are significant differences between them (*p* < 0.05).

**Figure 2:**
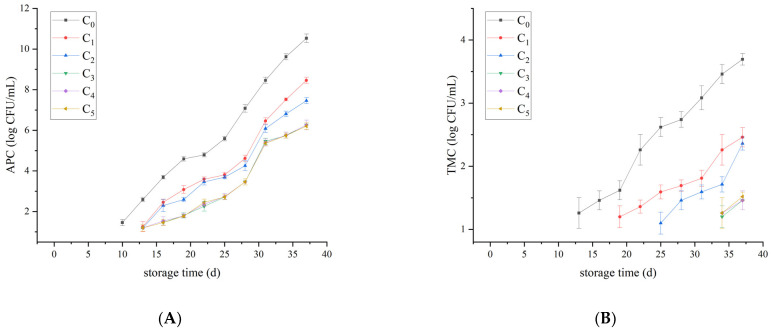

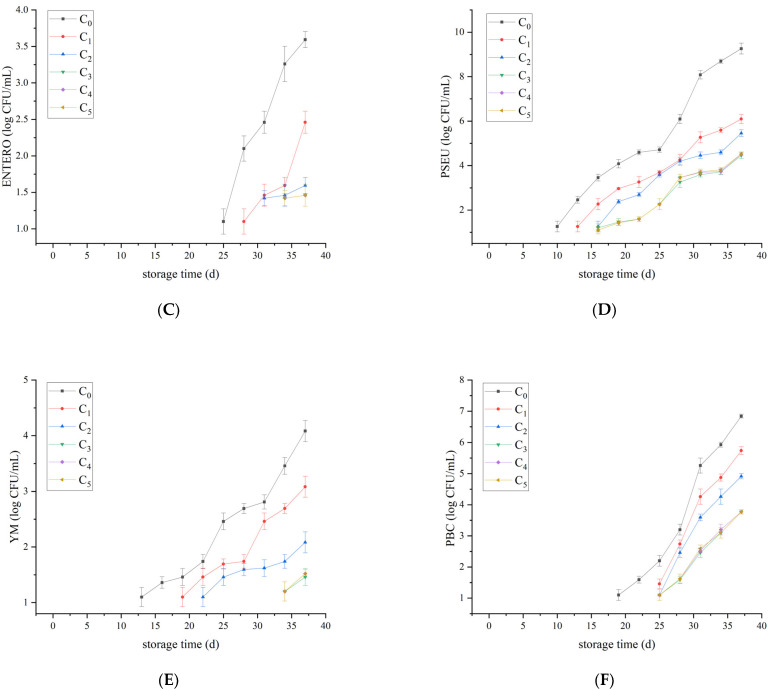
Microbiological counts of the pasteurized milk. (**A**) APC, (**B**) TMC, (**C**) ENTERO, (**D**) PSEU, (**E**) YM, and (**F**) PBC. Plantaricin FB-2 concentration was C_1_ = 0.1 g/kg, C_2_ = 0.2 g/kg, C_3_ = 0.3 g/kg, C_4_ = 0.4 g/kg, and C_5_ = 0.5 g/kg, and C_0_ was the control group, and the same amount of normal saline was added.

In the pasteurized milk (with a shelf life of 7 days), the national food safety standard of China stipulates that pasteurized milk APC is ≤1 × 10^5^ CFU/mL. As can be seen from Figure 2, no microbial vital activity was detected during its shelf life. On day 25, the APC in the control group was 3.89 × 10^5^ CFU/mL and began to deteriorate. On day 31, the APC in C_3_ group was 2.88 × 10^5^ CFU/mL. From day 10, *Pseudomonas* and mesophilic bacteria could be detected. On the last day of storage, the PSEU of the control group reached 1.82 × 10^9^ CFU/mL, while that of C_4_ group was only 3.31 × 10^4^ CFU/mL, and the differences were significant (*p* < 0.05). From day 13, the existence of YM was detected. From day 19, the presence of psychrotrophic bacteria could be detected. From day 25, the existence of *Enterobacteriaceae* could be detected. It is worth mentioning that throughout the storage period, *Staphylococcus aureus* was not detected.

In the comprehensive analysis of microbial changes during the whole storage period, it was found that in liquid milk, *Pseudomonas* was the dominant spoilage bacteria from the beginning to the end, while psychrotrophic bacteria gradually became the dominant spoilage bacteria in the late storage period. Psychrotrophic bacteria shows strong viability at low temperatures, they can produce proteolytic and lipolysis enzymes, cause various defects, and contaminate dairy products [23]. In addition, *Enterobacteriaceae* and *Staphylococcus aureus* were also dominant spoilage bacteria in raw milk. In pasteurized milk, mesophilic bacteria displayed strong vitality and became the dominant spoilage bacteria. Plantaricin FB-2 has a unique bacteriostatic ability, which can delay the deterioration of liquid milk. However, due to the high nutritional content of milk, the microbial flora during storage is very complex. Studies have reported the presence of lactic acid bacteria, *Acinetobacter* spp., *Streptococcus,* and *Aeromonas* spp. [24]. Anaerobic flora, including *Bacteroides*, *Faecalibacterium*, *Prevotella,* and *Catenibacterium* were also detected [3]. The role of the above bacteria in milk storage needs further exploration.

### 3.2. Microbial Model Analysis of Raw Milk

**Figure 3:**
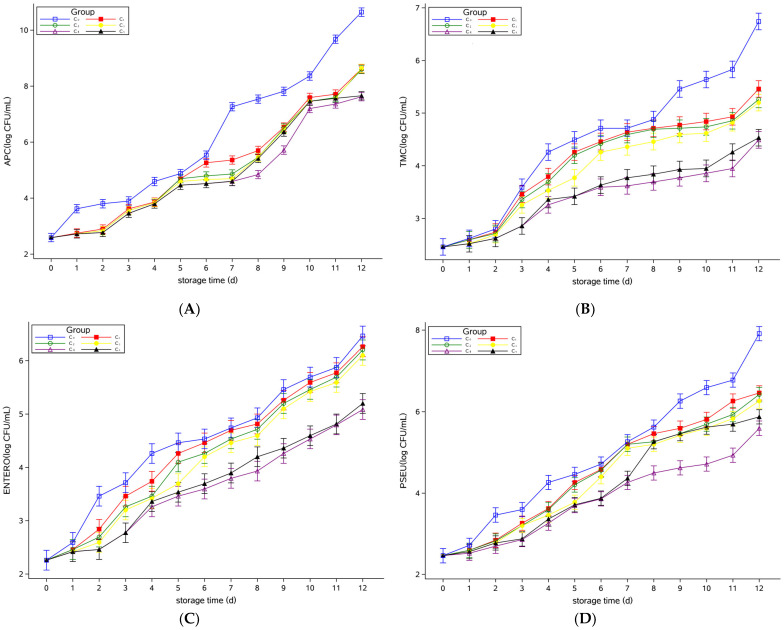

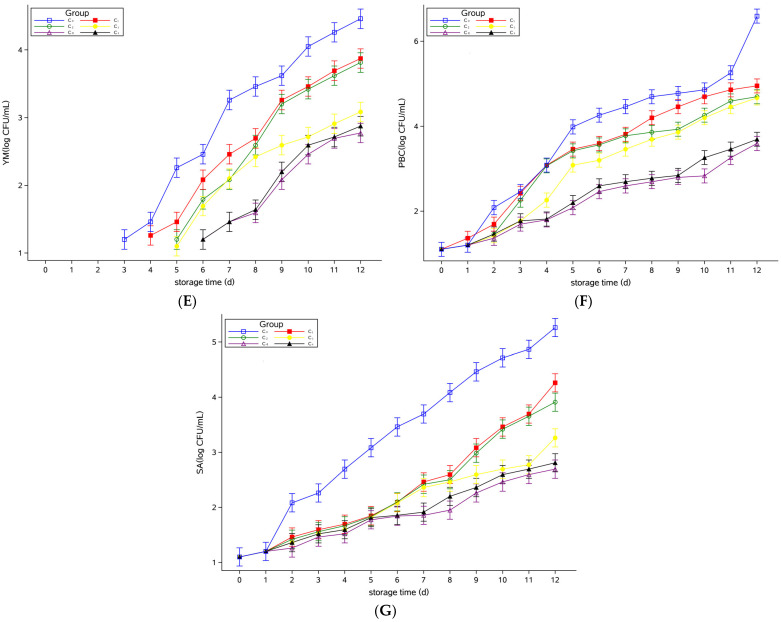
Least squares means of the logarithmic values of the number of colonies microorganism in the raw milk. (**A**) APC, (**B**) TMC, (**C**) ENTERO, (**D**) PSEU, (**E**) YM, (**F**) PBC, and (**G**) SA. Plantaricin FB-2 concentration was C_1_ = 0.1 g/kg, C_2_ = 0.2 g/kg, C_3_ = 0.3 g/kg, C_4_ = 0.4 g/kg and C_5_ = 0.5 g/kg, and C_0_ was the control group, and the same amount of normal saline was added.

Based on the determination of APC, TMC, ENTERO, PSEU, YM, PBC, and SA during the storage period of raw milk, the mixed effect model was carried out to analyze the bacterial levels in the groups supplemented with different concentrations of Plantaricin FB-2. Group C_0_ was the control group, and groups C_1_, C_2_, C_3_, C_4_, and C_5_ were the treatment group for days 0–12. The results were shown in Figure 3, representing the least squares mean of the logarithmic values of the number of colonies per microorganism, which were consistent with the trend in the measured values reported in Section 3.1. The confidence level was 95%. The results of significance analysis showed no significant difference among them.

Berhanu et al. [25] proved that the APC and ENTERO increased with time during storage and transportation of raw milk. Machado et al. [26] demonstrated that there are many kinds of microorganisms in raw milk, among which *Pseudomonas* was the dominant organism. As storage time increased, the microbial species were dominated by psychrotrophic bacteria. This was consistent with the results of this study that the microbial content of raw milk increased with storage time. In the later stages of storage, *Pseudomonas* aeruginosa and psychrotrophic bacteria were the main species.

### 3.3. Microbial Model Analysis of Pasteurized Milk

**Figure 4:**
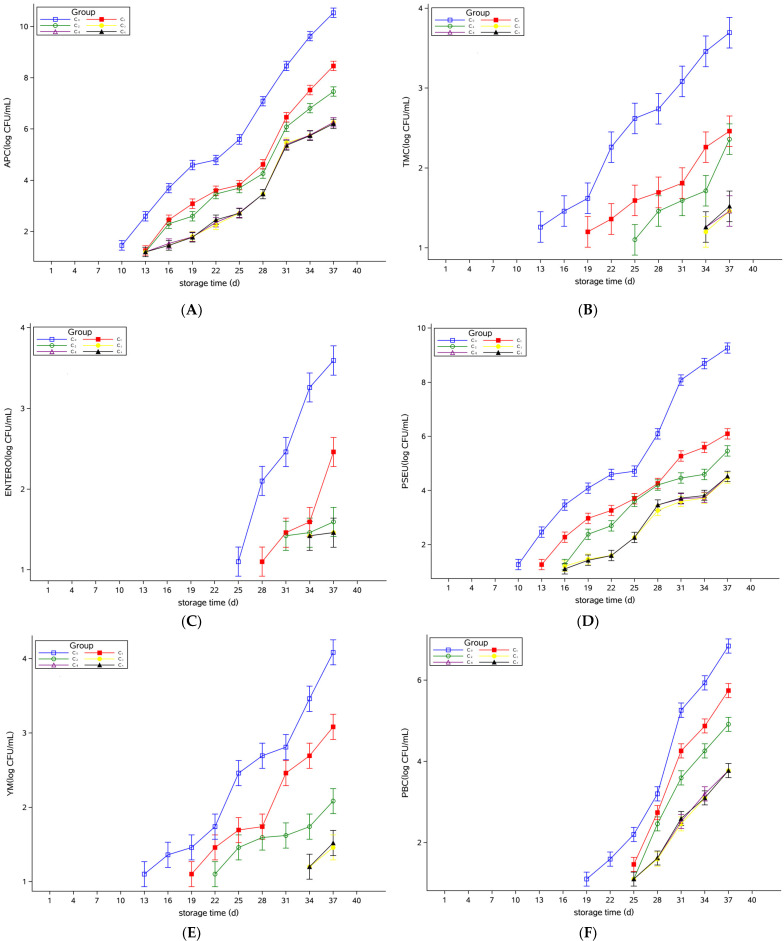
Least squares means of the logarithmic values of the number of colonies microorganism in the pasteurized milk. (**A**) APC, (**B**) TMC, (**C**) ENTERO, (**D**) PSEU, (**E**) YM, and (**F**) PBC. Plantaricin FB-2 concentration was C_1_ = 0.1 g/kg, C_2_ = 0.2 g/kg, C_3_ = 0.3 g/kg, C_4_ = 0.4 g/kg, and C_5_ = 0.5 g/kg, and C_0_ was the control group, and the same amount of normal saline was added.

Based on the determination of APC, TMC, ENTERO, PSEU, YM, and PBC during the storage period of pasteurized milk, the mixed effect model was used to analyze the bacterial levels of the groups supplemented with different concentrations of Plantaricin FB-2. The results were shown in Figure 4, indicating the least squares mean of the logarithmic values of the number of colonies per microorganism, which were consistent with the trend in the measured values reported in Section 3.1. The confidence level was 95%. The results of significance analysis showed no significant difference among them.

Yu et al. [27] demonstrated that pasteurization can effectively prolong the shelf life of raw milk. However, Ribeiro Júnior et al. [28] proved that bacteria can multiply at refrigerated temperatures, resulting in a shortened shelf life of pasteurized milk. Kable et al. [29] showed that *Pseudomonas* was the dominant spoilage agent in pasteurized milk. This was also confirmed in the present study. The PSEU was close to the APC on the last day of storage, which was sufficient evidence that *Pseudomonas* was the dominant spoilage organism in pasteurized milk.

### 3.4. Methylene Blue Reduction Reaction Analyses

The methylene blue reduction reaction method can be used to estimate the degree of bacterial contamination in milk. This is a simple and effective method to check milk before distribution. Fresh milk is dyed blue when methylene blue is added. If a large number of contaminated microorganisms produce reductase, the color gradually becomes lighter until invisible. By measuring the color change time, the hygienic quality of fresh milk can be inferred indirectly.

**Figure 5:**
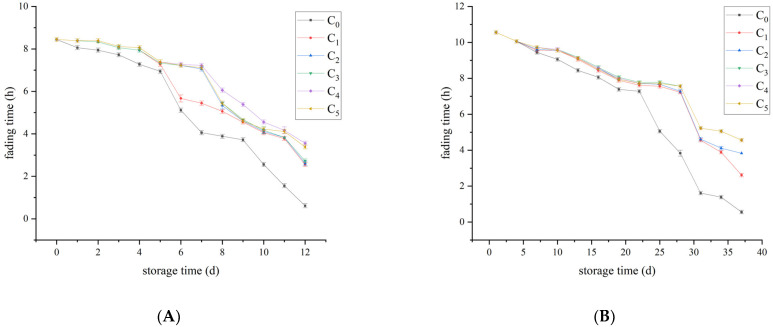
Fading time of methylene blue reduction reaction of the raw milk and pasteurized milk. (**A**) raw milk and (**B**) pasteurized milk.

It can be seen from Figure 5 that whether it is raw milk or pasteurized milk, the fading speed was accelerated with the prolongation of storage time. In raw milk, the fading time of the control group decreased sharply from day 5 onwards. From the 7th day, the fading time of the treatment group began to decline sharply. On day 12, the fading time of the control group was 36 min, while that of the C_4_ group was 3.5 h, and the difference between them was significant (*p* < 0.05). In the pasteurized milk, the fading time of the control group decreased sharply from day 22 onwards. From day 28, the time to fading in the treatment groups began to decline dramatically. In the end, the fading time of the control group was about 33 min, while that of the C_3_ group was 4.5 h, and the difference was significant (*p* < 0.05).

The results of the methylene blue reduction reaction were consistent with the results of microbial analysis. The longer the storage time, the faster the growth rate of microorganisms, leading to accelerated deterioration of raw and pasteurized milk, and the shorter the fading time of methylene blue reduction reaction. Meanwhile, the effect of Plantaricin FB-2 was dose- and time-dependent, and the fading time was slower in the high concentration group.

### 3.5. Acidity Analyses

**Figure 6:**
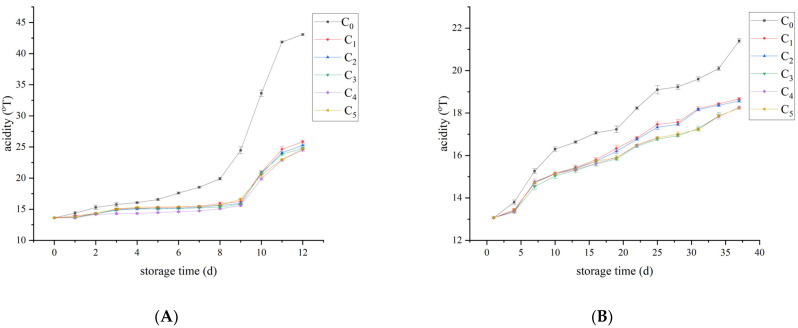
Changes in acidity of the raw milk and pasteurized milk. (**A**) raw milk and (**B**) pasteurized milk.

Increased acidity leads to sour odor in dairy products, which affects the sensory evaluation of dairy products. The initial acidities of raw milk and pasteurized milk were 13.6 °T and 13.1 °T respectively. From Figure 6, it can be seen that the overall acidity of both raw milk and pasteurized milk showed an increasing trend during storage. In the raw milk, from the 7th day, the acidity of the control group reached 18.53 °T and began to deteriorate. On the day 10, the acidity of C_4_ group increased sharply to 19.87 °T, while the acidity of the control group reached 33.63 °T, and the difference between the two groups was significant (*p* < 0.05). The acidity of control group was as high as 43.07 °T at the end of storage, while the acidity of the treatment group was between 24.67 °T and 25.83 °T. In pasteurized milk, the acidity in the control group reached 18.23 °T and started to deteriorate from day 22. On the 37th day, the C_3_ group began to deteriorate, and its acidity reached 18.23 °T, while that of the control group reached 21.40 °T, and the difference was statistically significant (*p* < 0.05).

Barbano et al. [30] proved that the average acid value of raw milk and pasteurized milk of low-SCC (somatic cell count) milk was significantly lower than that of high-SCC milk, and with the extension of storage time, the acid value of high-SCC milk was significantly higher than low-SCC milk. This is consistent with the results of this study. The change in acidity was mainly caused by the change in microorganism, which was basically consistent with the growth trend of the above microorganism. Acidity in unpasteurized milk increases significantly with the duration of refrigeration, probably due to the action of lipases, which was inactivated by pasteurization, delaying the rise in raw milk acidity [31]. This was also confirmed in our study, where acidity increased more slowly in pasteurized milk than in raw milk. Organic acids in the metabolites of acidifying bacteria (such as lactic acid bacteria) accelerate the acidification of milk, resulting in the increase in milk acidity and lower quality of raw and pasteurized milk [32].

### 3.6. Protein Content Analyses

**Figure 7:**
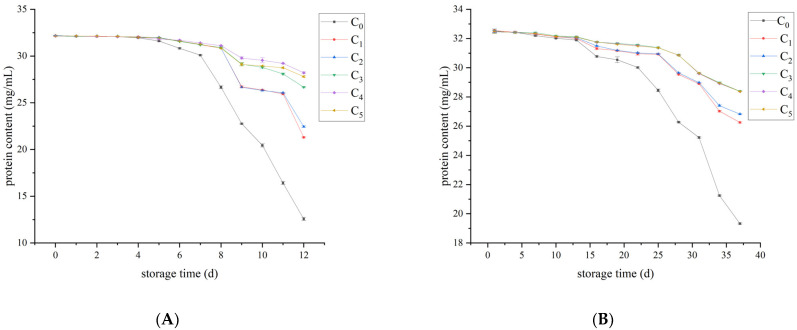
Changes in protein content of the raw milk and pasteurized milk. (**A**) raw milk and (**B**) pasteurized milk.

Protein is an important nutrient in milk, and its content can directly reflect the quality of liquid milk. As can be seen from Figure 7, the protein content of raw and pasteurized milk generally showed a downward trend during storage. The addition of Plantaricin FB-2 can delay the decline of protein content in a concentration-dependent manner. In raw milk, the protein content of the samples without Plantaricin FB-2 began to decrease sharply after 7 days, but Plantaricin FB-2 can delay the rate of protein content decline. There was a significant difference between C_4_ group and other groups (*p* < 0.001), so the best preservation effect was obtained when the added amount was 0.4 g/kg. In pasteurized milk, the protein content of the control group began to decrease sharply after 22 days, and Plantaricin FB-2 can delay the rate of protein content decline. The differences among C_3_, C_4_, and C_5_ groups were not significant, but compared with other groups, the difference was extremely significant (*p* < 0.001), so the preservation effect was the best when the supplemental amount was 0.3~0.5 g/kg. Considering the cost factor, it was considered that the optimal supplemental amount is 0.3 g/kg.

Studies have demonstrated that milk with high SCC undergoes more extensive casein degradation than milk with low SCC. In particular, high-SCC milk showed that significant proteolytic activity remained after pasteurization. High-SCC raw milk has high concentrations of somatic cell derived plasminogen, plasminogen, and protease. Plasminase is thermostable and survives minimum pasteurization (72 °C/15 s) in large quantities. Even after UHT treatment, 30–40% of the plasminase activity is retained [30]. In our study, the protein degradation rate of raw milk was significantly higher than that of pasteurized milk. When milk was stored, microorganisms continued to grow and reproduce, which may decompose proteins and cause protein loss. The most dominant microorganisms were psychrotrophic bacteria, among which *Pseudomonas* was the most common. A specific extracellular alkaline metalloproteinase belonging to the AprX enzyme family has been found in *Pseudomonas*. This enzyme can cause proteolysis, resulting in a reduction in protein content [33,34]. The combination of microbial changes and acidity changes show that microbial life activities lead to an increase in milk acidity and protein degradation, and a decrease in protein concentration. The increasing trend of spoilage microorganisms is basically consistent with milk acidification and protein loss. Protein content is an important nutritional indicator of dairy products. The loss of protein affects the nutritional value of liquid milk, and whey stratification affects the sensory value of liquid milk.

### 3.7. Sensory Evaluation Analyses

Sensory quality is an important quality of products. The United States have taken sensory indicators as the main basis for food safety warnings, deterioration, and adulteration inspections. The sensory quality of milk is an important factor affecting the market acceptance of milk products and consumer purchasing behavior [35]. In our study, the sensory scores showed an overall downward trend with increasing storage time.

**Figure 8:**
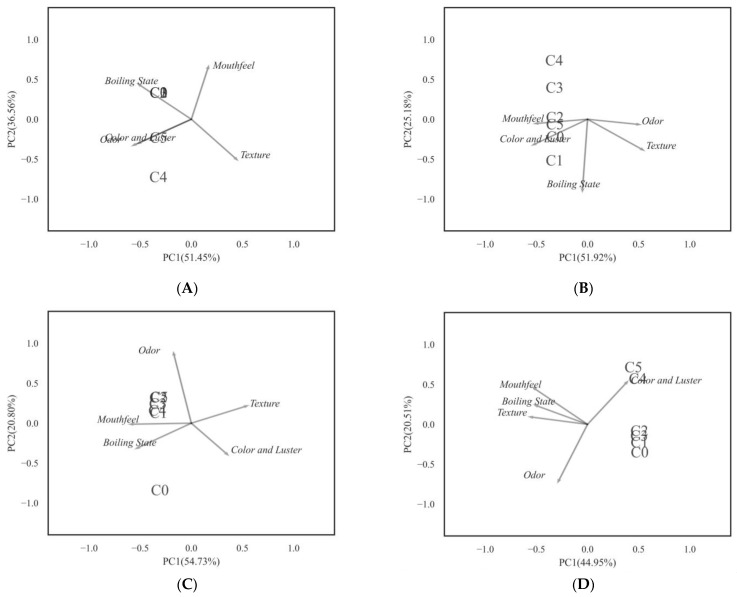
PCA factor map of the descriptive analysis data in raw milk. (**A**) day 3, (**B**) day 6, (**C**) day 9 and, (**D**) day 12. Plantaricin FB-2 concentration was C_1_ = 0.1 g/kg, C_2_ = 0.2 g/kg, C_3_ = 0.3 g/kg, C_4_ = 0.4 g/kg, and C_5_ = 0.5 g/kg, and C_0_ was the control group, and the same amount of normal saline was added.

In raw milk, as shown in Table 3, the effect of texturing was significant at day 12, with no other significant differences. It was noteworthy that the C_5_ scores for each indicator were lower than C_4_. PCA applied to the DA data table accounted for 65.46% of the variance in the first two dimensions. It can be seen from Figure 8D that the sensory evaluation of the high-concentration groups (C_4_ and C_5_) was mainly influenced by color and luster, while the other groups were mainly influenced by both odor and color and luster.

**Figure 9:**
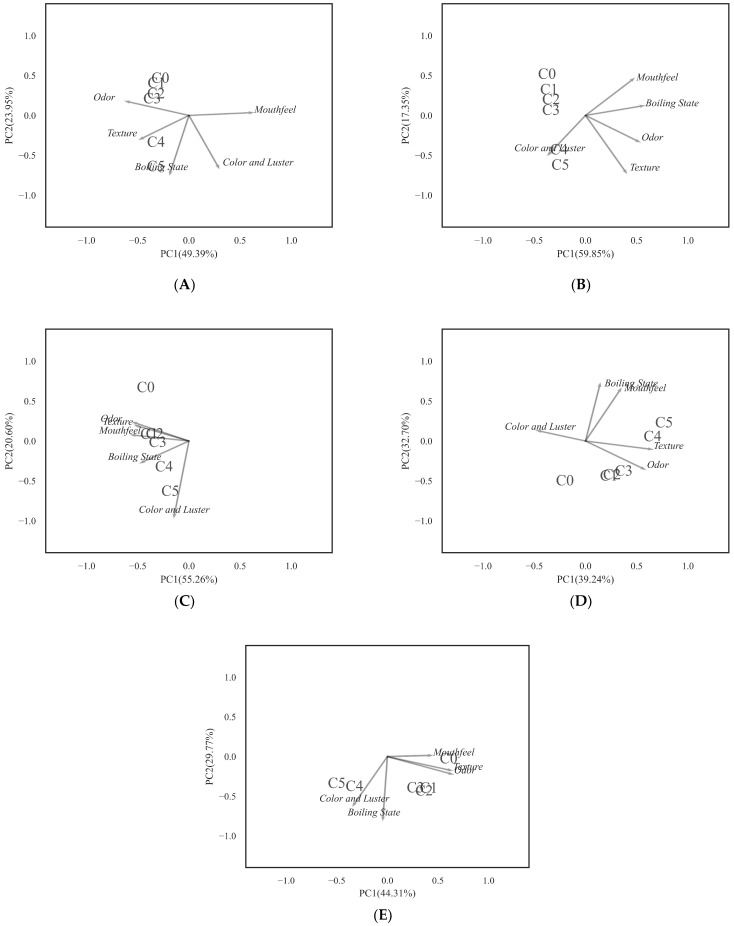
PCA factor map of the descriptive analysis data in pasteurized milk. (**A**) day 13, (**B**) day 19, (**C**) day 25, (**D**) day 31, and (**E**) day 37. Plantaricin FB-2 concentration was C_1_ = 0.1 g/kg, C_2_ = 0.2 g/kg, C_3_ = 0.3 g/kg, C_4_ = 0.4 g/kg, and C_5_ = 0.5 g/kg, and C_0_ was the control group, and the same amount of normal saline was added.

In pasteurized milk, as shown in Table 4, the effect of texturing was significant at days 25, 31, and 37, with no other significant differences. The color and luster of the high-concentration groups (C_4_ and C_5_) were lower than that of other groups. PCA applied to the DA data table accounted for 74.08% of the variance in the first two dimensions. It can be seen from Figure 9E that the sensory evaluation of the high-concentration groups (C_4_ and C_5_) was mainly influenced by color and luster and boiling state, and the low-concentration groups (C_1_, C_2_ and C_3_) were mainly influenced by both odor and boiling state. The control group quality was affected by mouthfeel, texture, and odor.

It has been found that the addition of bioactive peptides enhanced the color parameters and sensory properties of milk [36]. This is consistent with the conclusion of this study that the addition of Plantaricin FB-2 delayed the spoilage of milk, thus improving the sensory properties. Nevertheless, due to whey separation in liquid milk at the post-storage stage, Plantaricin FB-2 was precipitated, which seriously affected the color and luster evaluation of C_5_ group. This also suggested that the amount of food preservatives should be appropriate and selected in consideration of the food’s sensory indicators. Overall, microbial life activities, acidity increase, and protein degradation can affect the sensory evaluation of milk, resulting in sour odor, whey separation, and poor tissue status, thereby reducing consumers’ desire to buy.

## 4. Conclusions

For raw milk, when the supplemental level of Plantaricin FB-2 was 0.4 g/kg, the shelf life of raw milk was extended by 3 days. For pasteurized milk, the addition of 0.3 g/kg of Plantaricin FB-2 worked best, extending the shelf life of pasteurized milk by 6 days. Especially in the microbial analysis, the mixed effects models were used for microbial prediction. This study confirmed the fresh-keeping effect of Plantaricin FB-2 on liquid milk and provided biological preservative for prolonging the shelf life of raw milk and pasteurized milk. In future research, Plantaricin FB-2 can be applied as a natural preservative in other foods, broadening its application range.

## Figures and Tables

**Table 1:** Microorganism culture method.

Microorganism	Culture Medium	Culture Condition
APC	PCA	30 °C, 48–72 h
TMC	MPCA	30 °C, 48 h
ENTERO	VRBGA	30 °C, 24 h
PSEU	*Pseudomonas* CFC selective agar + 5 mL glycerol + CFC supplements	30 °C, 48 h
YM	DRBC	28 °C, 5 d
PBC	MPC	6.5 °C, 10 d
SA	Baird-Parker	36 °C ± 1 °C, 24–48 h

Note: APC: aerobic plate count; TMC: total mesophilic count; ENTERO: *Enterobacteriaceae* selective count; PSEU: *Pseudomonas* selective count; YM: yeast and mold; PBC: psychrotrophic bacterial count; SA: *Staphylococcus aureus*.

**Table 2:** Sensory evaluation standard for liquid milk.

Evaluation Index	Standard	Grade
Odor	With milk inherent fragrance, no peculiar smell	16–20
	Slight fragrance, fragrance is not obvious	11–15
	A slight odor	6–10
	Odor obvious	0–5
Color and luster	Milky white or yellowish	16–20
	The color begins to darken to yellow	11–15
	The color is dull and burnt yellow	6–10
	Pale blue or other unusual colors	0–5
Texture	Uniform fluid, no clots, no precipitation, no normal vision of foreign bodies	16–20
	Slightly viscous, uneven liquid	11–15
	Viscous, slightly layered	6–10
	Stratification is serious, the upper layer appears clear liquid, the lower layer appears bean curd-like sediment	0–5
Boiling state	Liquid evenly	16–20
	Slight flocculent solidification begins	11–15
	Semi-solidified state	6–10
	Completely frozen	0–5
Mouthfeel	The characteristic mellow taste of milk	16–20
	The fragrance is light, but no peculiar smell	11–15
	The appearance of atypical frankincense or sour taste	6–10
	Acid smell	0–5

**Table 3:** Overall means and LSD values for the significant descriptive analysis attributes (*p* < 0.05). Plantaricin FB-2 concentration was C_1_ = 0.1 g/kg, C_2_ = 0.2 g/kg, C_3_ = 0.3 g/kg, C_4_ = 0.4 g/kg, and C_5_ = 0.5 g/kg, and C_0_ was the control group, and the same amount of normal saline was added.

Day	Milk	Odor		Color and Luster		Texture		Boiling State		Mouthfeel	
0	C_0_	19.4	a ^*a*^	19.6	a	19.8	a	20	a	19.8	a
	C_1_	19.6	a	19.6	a	19.8	a	20	a	19.8	a
	C_2_	19.6	a	19.6	a	19.8	a	20	a	19.8	a
	C_3_	19.6	a	19.6	a	19.8	a	20	a	19.8	a
	C_4_	19.6	a	19.6	a	19.8	a	20	a	19.8	a
	C_5_	19.6	a	19.6	a	19.8	a	20	a	19.8	a
	LSD	0.71		0.71		0.58		0.00		0.58	
3	C_0_	17.8	a	17.6	a	18.2	a	17.8	a	17.2	a
	C_1_	17.8	a	17.6	a	18.2	a	17.8	a	17.2	a
	C_2_	17.8	a	17.6	a	18.2	a	17.8	a	17.2	a
	C_3_	17.8	a	17.6	a	18.2	a	17.8	a	17.2	a
	C_4_	18	a	17.6	a	18.4	a	17.8	a	17.2	a
	C_5_	17.8	a	17.4	a	18.2	a	17.8	a	17.2	a
	LSD	1.07		1.11		0.61		0.58		0.58	
6	C_0_	13.2	a	13	a	13.6	a	13.4	a	12.4	a
	C_1_	13.4	a	13.2	a	13.6	a	13.6	a	12.4	a
	C_2_	13.4	a	13	a	13.6	a	13.4	a	12.6	a
	C_3_	13.4	a	13	a	13.6	a	13.6	a	12.8	a
	C_4_	13.4	a	13	a	13.8	a	13.8	a	13	a
	C_5_	13.4	a	12.8	a	13.6	a	13.6	a	12.4	a
	LSD	0.69		0.83		0.69		0.69		0.73	
9	C_0_	8.2	b	9.2	a	9.4	a	8.6	a	8.6	a
	C_1_	9.2	a	9.2	a	9.4	a	8.6	a	8.6	a
	C_2_	9.4	a	9.2	a	9.4	a	8.6	a	8.6	a
	C_3_	9.4	a	9.2	a	9.4	a	8.8	a	8.8	a
	C_4_	9.4	a	9.2	a	9.8	a	8.8	a	8.8	a
	C_5_	9.2	a	8.8	a	9.4	a	8.4	a	8.6	a
	LSD	0.65		0.58		0.69		0.67		0.67	
12	C_0_	3.4	a	7.2	a	3.4	b	3.6	a	3.4	a
	C_1_	3.6	a	7.2	a	3.4	b	3.6	a	3.4	a
	C_2_	3.6	a	7.2	a	3.6	ab	3.6	a	3.4	a
	C_3_	3.6	a	7.2	a	3.6	ab	3.8	a	3.4	a
	C_4_	4.2	a	7.2	a	4.2	a	4	a	3.8	a
	C_5_	4	a	6.6	a	4	ab	3.4	a	3.4	a
	LSD	0.83		0.61		0.63		0.63		0.69	

^*a*^ In columns, means sharing the same letter are not significantly different.

**Table 4:** Overall means and LSD values for the significant descriptive analysis attributes (*p* < 0.05). Plantaricin FB-2 concentration was C_1_ = 0.1 g/kg, C_2_ = 0.2 g/kg, C_3_ = 0.3 g/kg, C_4_ = 0.4 g/kg, and C_5_ = 0.5 g/kg, and C_0_ was the control group, and the same amount of normal saline was added.

Day	Milk	Odor		Color and Luster		Texture		Boiling State		Mouthfeel	
1	C_0_	20	a ^*a*^	20	a	20	a	20	a	19.8	a
	C_1_	20	a	20	a	20	a	20	a	19.8	a
	C_2_	20	a	20	a	20	a	20	a	19.8	a
	C_3_	20	a	20	a	20	a	20	a	19.8	a
	C_4_	20	a	19.4	b	20	a	20	a	19.8	a
	C_5_	20	a	19.2	b	20	a	20	a	19.6	a
	LSD	0.00		0.38		0.00		0.00		0.61	
7	C_0_	19.8	a	19.6	a	19.8	a	20	a	19.2	a
	C_1_	19.8	a	19.6	a	19.8	a	20	a	19.2	a
	C_2_	19.8	a	19.6	a	19.8	a	20	a	19.2	a
	C_3_	19.8	a	19.6	a	19.8	a	20	a	19.2	a
	C_4_	19.8	a	19.2	a	19.8	a	20	a	19.2	a
	C_5_	19.8	a	19	a	19.8	a	20	a	19.2	a
	LSD	0.58		0.83		0.58		0.00		1.09	
13	C_0_	17.6	a	17.8	a	18.2	a	17.8	a	16.6	a
	C_1_	17.6	a	17.8	a	18.4	a	17.8	a	16.6	a
	C_2_	17.8	a	17.8	a	18.4	a	17.8	a	16.6	a
	C_3_	17.8	a	17.8	a	18.6	a	17.8	a	16.6	a
	C_4_	17.8	a	17.4	a	18.4	a	17.8	a	16.6	a
	C_5_	17.8	a	17.2	a	18.4	a	17.8	a	16.6	a
	LSD	0.63		0.71		0.69		0.58		1.17	
19	C_0_	13.6	a	15.2	a	12.8	a	13.2	a	11.2	a
	C_1_	13.8	a	15.2	a	13.2	a	13.4	a	11.4	a
	C_2_	13.8	a	15	a	13.2	a	13.4	a	11.4	a
	C_3_	13.8	a	15	a	13.4	a	13.6	a	11.4	a
	C_4_	13.8	a	14.2	b	13.4	a	13.6	a	11.4	a
	C_5_	14	a	14	b	13.4	a	13.6	a	11.4	a
	LSD	1.01		0.77		0.65		0.69		0.79	
25	C_0_	11.6	b	12.8	a	11.4	b	11.8	a	10.4	b
	C_1_	12.4	a	12.8	a	12	ab	12.2	a	10.8	ab
	C_2_	12.4	a	12.8	a	12.2	a	12.2	a	11	ab
	C_3_	12.6	a	12.8	a	12.2	a	12.4	a	11.2	a
	C_4_	12.6	a	12.4	a	12.2	a	12.4	a	11.2	a
	C_5_	12.6	a	12	a	12.2	a	12.4	a	11.2	a
	LSD	0.71		1.01		0.67		0.92		0.67	
31	C_0_	6.4	b	7.4	a	6.2	b	6.8	a	6.2	a
	C_1_	7.6	a	7.4	a	7.2	ab	7.2	a	6.6	a
	C_2_	7.6	a	7.4	a	7.4	ab	7.2	a	6.6	a
	C_3_	8	a	7.4	a	7.6	a	7.2	a	6.8	a
	C_4_	8	a	6.4	b	7.6	a	7.2	a	6.8	a
	C_5_	8	a	6	b	7.6	a	7.2	a	6.8	a
	LSD	0.83		0.75		1.26		1.09		0.91	
37	C_0_	4.2	b	6	a	4.2	b	5.2	ab	4.2	a
	C_1_	5.6	a	6	a	4.6	ab	5.8	a	4.4	a
	C_2_	5.8	a	6	a	4.8	ab	5.8	a	4.4	a
	C_3_	6	a	6	a	5.2	a	5.8	a	4.6	a
	C_4_	6	a	3.8	b	5.2	a	5.2	ab	4.6	a
	C_5_	6	a	3.2	b	5.2	a	4.8	b	4.6	a
	LSD	0.79		0.83		0.61		0.88		0.69	

^*a*^ In columns, means sharing the same letter are not significantly different.

## Data Availability

Data is contained within the article.
